# Associations between Parental Feeding Styles and Childhood Eating Habits: A Survey of Hong Kong Pre-School Children

**DOI:** 10.1371/journal.pone.0124753

**Published:** 2015-04-30

**Authors:** Kenneth Lo, Calvin Cheung, Albert Lee, Wilson W. S. Tam, Vera Keung

**Affiliations:** 1 JC School of Public Health and Primary Care, Faculty of Medicine, Shatin, The Chinese University of Hong Kong, Hong Kong, Hong Kong; 2 Centre for Health Education and Health Promotion, Shatin, The Chinese University of Hong Kong, Hong Kong, Hong Kong; 3 Alice Lee Centre for Nursing Studies, Yong Loo Lin School of Medicine, National University of Singapore, Singapore, Singapore; National Research Council (CNR), ITALY

## Abstract

Childhood obesity is a global public health issue, including in the Chinese setting, and its prevalence has increased dramatically throughout the last decade. Since the origins of childhood obesity may lie in the pre-school period, factors relating to very young children’s food consumption should be investigated. Parental influence, including feeding style, is the major determinant of childhood dietary behaviour through altering food provision and social environment. However, the applicability of previous research on parental feeding styles was limited by small sample size. To evaluate the influence of parental feeding styles on children's dietary patterns, a cross-sectional study was conducted among 4553 pre-schoolers in Hong Kong. Information was obtained about dietary intake and how regularly they had breakfast, using previous health surveillance surveys taken among primary school students. Parental feeding styles were assessed by a validated Parental Feeding Style Questionnaire and categorized into ‘instrumental feeding’, ‘emotional feeding’, ‘prompting and encouragement to eat’ and ‘control over eating’. Multivariable analysis was performed, adjusted for demographic information. Instrumental and/or emotional feeding was found to relate to inadequate consumption of fruit, vegetables and breakfast, and positively correlated with intake of high-energy-density food. Encouragement on eating was associated with more frequent consumption of fruits, vegetables, dairy products and breakfast. Control over eating correlated with more frequent consumption of fruits, vegetables and breakfast, and less consumption of dairy products and high-energy-density food. The present study has provided evidence on the associations between parental feeding styles and dietary patterns of Hong Kong pre-school children from a reasonably large population. Parents should avoid instrumental and emotional feeding, and implement control and encouragement to promote healthy food intake. Longitudinal studies and interventions on parental feeding style are required to confirm the research findings.

## Introduction

Childhood obesity is a major public health issue worldwide, including among the Chinese. The prevalence of childhood obesity among Chinese children has increased dramatically from 4.1% in 2000 to 8.1% in 2010.[1] The severity of childhood obesity in Hong Kong is similar to that in China. According to statistics provided by the Hong Kong Department of Health, the prevalence of childhood obesity among primary one students rose from 11.3% in school year 1996–97 to 15.3% in 2010–11.[2] This result indicated that childhood obesity might originate in the pre-school period. To prevent various obesity-related chronic diseases in later stages of life, it is necessary to teach children healthier eating habits.

The Hong Kong Department of Health has recommended that children aged 2–6 consume one serving of fruits, two to three servings of vegetables and two servings of dairy products and minimal fat or sugar per day.[3,4] Adequate intake of fruit, vegetables and/or dairy products is linked to lower incidence of chronic diseases in later life, [5,6] whereas high intake of high-fat and sugar-rich foods with low nutrient levels, known as high-energy-density (high-ED) foods, may lead to sub-optimal intake of the recommended food groups among pre-school children, as observed from a local survey.[7] Additionally, missing breakfast is a well-known cardio-metabolic risk factor.[8] Given the importance of the above dietary practices, the promotion of good health should focus on them.

Young children have limited autonomy and they are dependent on adult supervision. Therefore, adult parents or guardians may affect children’s dietary patterns by influencing food provision and social environment.[9] Parental feeding styles, referring to the specific techniques or behaviours that influence children’s dietary patterns,[10] have demonstrated associations between parental feeding styles and children's fruit and vegetable intake,[11,12] high fat and/or sugar intake,[13] dairy intake,[14] and weight.[15]

Despite the above influence on children's food consumption, interpretation of research findings can be difficult. Parental feeding styles represent multi-dimensional psychometric measures; they can be categorized by demandingness and responsiveness into authoritative, authoritarian, and permissive styles, which are closely related to parenting styles.[16] They can also be classified by levels of self-efficacy, consistency, over-protectiveness and many others.[17] Various instruments have been developed for assessing the dimensions of parenting feeding styles:[18] the most widely used tool is the Child Feeding Questionnaire, [17,19] and its scales for assessing ‘controlling feeding practice’ have been used by numerous studies. [20] Aside from parental control over eating, research on other constructs of feedings styles are inadequate.

The Parental Feeding Style Questionnaire (PFSQ) is a 27-item questionnaire for assessment of four scales, namely ‘Instrumental Feeding’ (feeding children in response to their behaviour), ‘Emotional Feeding’ (feeding children in response to their emotions), ‘Prompting and Encouragement to eat’ (encouraging children to consume a variety of foods), and ‘Control over Eating’ (determining the types and quantities of foods that children should consume).[21] From studies conducted among parents and children aged 6–7 years, instrumental feeding in mothers was positively associated with eating initiated by smell and appearance [21] and children’s consumption of snack. [22] Emotional feeding was also related to more frequent snacking behaviour in children, while ‘Prompting and Encouragement to eat’ was negatively associated with children’s snack intake.[22]

Although parental feeding styles may relate to children’s dietary behaviour, this relationship has not been well established, due to relatively small sample sizes (less than 500 participants in each study). Research into how parental feeding styles can influence children’s dietary pattern before the age of six is lacking. Besides, food-related parenting practices have shown variations with demographic characteristics.[23] Given the demographic differences between China and western countries, a large-scale study needs to be conducted in a Chinese context. Research findings may verify results seen in previous literature, especially for instrumental and emotional feeding, which are worthy of more attention from researchers.

## Materials and Methods

A cross-sectional survey was conducted among Hong Kong pre-schoolers in December 2010. Letters of invitation were sent to 100 kindergartens (for students aged 2–5) in Hong Kong, which had participated in a health-promoting school project conducted by the Center for Health Education and Health Promotion of the Chinese University of Hong Kong. Twenty-seven kindergartens agreed to participate in the study and parents, who joined the study on a voluntary basis, were sent self-administered questionnaires with a consent form on the front page. The questionnaire was based on a questionnaire used for health surveillance among primary school students, [24] which was refined by nutrition experts to fit the local context of pre-school children. Questionnaires were to be completed by the children's parents or persons who had a good knowledge of their dietary intake.

The questionnaire included items assessing dietary intake seven days prior to the survey, breakfast-eating pattern, parental feeding style and demographic information about parents and children. Questions concerning consumption of fruit, vegetables and dairy products, as well as breakfast-eating pattern, were single-item questions with multiple choice answers. Questions were also asked about intake of commonly consumed high-ED food items such as sweets, soft drinks and sugary drinks. Reponses to questions ranged from ‘did not eat any fruit’ to ‘two or more servings of fruit per day’ for fruits, ‘did not eat any vegetables’ to ‘more than one bowl of vegetables per day’ for vegetables, ‘did not have or can’t have dairy products’ to ‘four or more servings per day’ for dairy products, ‘had breakfast everyday’ to ‘0 days’ for breakfast-eating pattern, and ‘0 times’ to ‘once or more per day’ for high-ED foods.

To facilitate valid input of intake, photographs were included to show the portion sizes for fruit, vegetables and dairy products, as well as common high-ED foods. Completed questionnaires were collected from kindergartens within two weeks of distribution, before data coding and analysis. Dietary data were categorized by the recommendation of specific food items. The frequency with which breakfast was eaten was differentiated into having breakfast every day or not. There is no recommended intake for high-ED foods, so the consumption of those food items was represented as continuous variables for data analysis. (‘0 times’ = 0; ‘1–2 times in 7 days’ = 1.5 divided by 7; ‘3–5 times in 7 days’ = 4 divided by 7; ‘once or more per day’ = 1). The average weekly intake for overall high-ED food items was derived from the quantity of each food item a child consumed.

A 27-item PFSQ [21] was used to assess parental feeding styles including ‘Instrumental Feeding’ (5 items), ‘Emotional Feeding’ (4 items), ‘Prompting and Encouragement to eat’ (8 items), and ‘Control over Eating’ (10 items). Respondents were asked to choose from a 5-point Likert scale (ranging from 'never' to 'always'). Average scores on each scale were calculated, whereby a higher score indicated a greater tendency for parents to feed their children in that style. A translation–back-translation procedure [25] was applied to create a Chinese version. The completed questionnaires had been pre-tested in a convenience sample of 10 parents from a kindergarten. The Chinese version of the PFSQ had also been validated as described previously.[26] The Cronbach’s alphas for instrumental feeding, emotional feeding, prompting or encouragement to eat, and control over eating were 0.63, 0.81, 0.83, and 0.63, respectively. The overall Cronbach’s alpha for all 27 items was 0.75. The identified factor structure in the present study was similar to the results in the Netherlands [22] and Costa Rica. [27]

Categorical variables included demographic information on parents and children, and their dietary intake of fruits, vegetables and dairy products. For continuous variables, the average overall value for weekly high-ED food consumption and the scores for each type of parental feeding style were computed. Multivariable logistic regression was performed to examine the impact of parental feeding styles on fulfilling recommended dietary intake or breakfast-eating pattern. Associations between parental feeding styles and consumption of high-ED food were investigated by multivariable linear regression. Unstandardized coefficients (β) with 95% confidence interval (95% C.I.), and odds ratio (OR) with 95% C.I. were computed for linear and logistic regressions, respectively, both adjusted for demographic information on parents and children. All results marked with an asterisk had a P-value lower than 0.05. PASW Statistics 17.0 software was used to for all data entry and analysis (SPSS Inc., Chicago, IL, USA).

## Results

Demographic information on children and parents is set out in Table 1. A total of 6186 questionnaires were distributed and 4553 were returned (73.6% response rate). Of the children, 51.8% were male. Only a very few children were attending nursery (age 2) (1.9%), while the distribution was similar for grade 1 (age 3) (34.5%), grade 2 (age 4) (31.9%) and grade 3 (age 5) (32.2%). Over half of the children (52.7%) attended half-day school in the morning session only, while most (93.8%) were born in Hong Kong; 45.4% children had one sibling and 37.3% were only children. For parents, the majority of fathers were full-time employees (84.0%), while nearly half of mothers were unemployed (48.8%). Most fathers (67.9%) and mothers (71.9%) had received secondary education. The monthly incomes of over half (61.9%) of the participating families were below HK$20,000. Most of the questionnaires (82.3%) were filled in by the children’s mothers.

**Table 1:** Demographic information of children and parents.

Characteristic	Frequency (%)
*Children*	
Gender	
Male	2360 (51.8%)
Female	2175 (47.8%)
Grade	
Nursery	59 (1.3%)
Grade 1	1572 (34.5%)
Grade 2	1453 (31.9%)
Grade 3	1468 (32.2%)
School status	
Whole day	777 (17.1%)
Half-day (a.m. only)	2400 (52.7%)
Half-day (p.m. only)	1369 (30.1%)
Place of birth	
Hong Kong	4270 (93.8%)
China	210 (4.6%)
Other place	47 (1.0%)
Siblings	
0	1700 (37.3%)
1	2069 (45.4%)
2 or more	743 (16.3%)
*Parents*	
Father’s job status	
Full-time	3823 (84.0%)
Part-time	278 (6.1%)
Unemployed	129 (2.8%)
Retired	27 (0.6%)
Not applicable/ Unknown	136 (3.0%)
Mother’s job status	
Full-time	1558 (34.2%)
Part-time	510 (11.2%)
Unemployed	2223 (48.8%)
Retired	9 (0.2%)
Not applicable/ Unknown	82 (1.8%)
Father’s education level	
Primary school or below	304 (6.7%)
Secondary school	3092 (67.9%)
Diploma/ Degree or higher	963 (21.2%)
Not applicable / Unknown	90 (2.0%)
Mother’s education level	
Primary school or below	378 (8.3%)
Secondary school	3272 (71.9%)
Diploma/ Degree or higher	790 (17.4%)
Not applicable / Unknown	50 (1.1%)
Household income per month	
<$10,000	946 (25.1%)
$10,000-$19,999	1389 (36.8%)
$20,000-$29,999	701 (18.6%)
$30,000-$39,999	354 (9.4%)
$40,000-$59,999	259 (6.9%)
≥$60,000	125 (3.3%)
Relationship to the child	
Father	580 (12.7%)
Mother	3748 (82.3%)
Others	165 (3.6%)
Parental feeding style	Average scores (standard deviation)
Instrumental feeding	2.68 (0.66)
Emotional feeding	2.31 (0.70)
Prompting and Encouragement to Eat	3.67 (0.64)
Control over eating	3.82 (0.50)

Parents were more likely to exercise ‘control over eating’ (3.87 marks) in feeding practice; ‘emotional feeding’ had the lowest tendency to be implemented (2.31 marks). As described in another published paper [7], the demographic characteristics of parents in this study were similar to those of the population of Hong Kong, which indicated the general applicability of this survey.

The proportions of children conforming to their recommended dietary intake and meal practices are described in Table 2. Only 47.3%, 19.6%, and 37.3% of pre-school children consumed the suggested intakes of fruits, vegetables and dairy products, respectively. Only 78.8% children did not skip breakfast in the 7 days prior to the survey. As shown in Table 3, average weekly intake of high-ED food ranged from less than 0.90 to 2.90 times per week, and the overall intake was 1.85 times per week.

**Table 2:** Number and percentage of children fulfilling recommended dietary intake and meal practice.

Food consumption or meal practice		Number (percentage)
Fruits (1 serving or more)	Yes	2293 (47.3%)
	No	2058 (52.7%)
Vegetables (2 servings or more)	Yes	850 (19.6%)
	No	3478 (80.4%)
Dairy products (2 servings or more)	Yes	1655 (37.3%)
	No	2778 (62.7%)
Breakfast (eat everyday)	Yes	3501 (78.8%)
	No	940 (21.2%)

**Table 3:** Average weekly intake of high-ED food (in number of times).

High-ED food (number of children)	Mean ± standard deviation
Candy or chocolate (4476)	2.90 ± 2.01
Chips and crisps (4439)	1.36 ± 1.50
Ice-cream, ice lolly (4417)	0.90 ± 1.27
Cake or tart (4449)	2.31 ± 1.78
Sweet cracker (4428)	2.09 ± 1.77
Stuffed bread roll or pastry (4440)	2.21 ± 1.86
Soda drinks (4462)	1.13 ± 1.54
Sugary drinks (4483)	2.17 ± 1.85
Deep-fried food (4465)	1.39 ± 1.24
Processed meat or cured meat (4470)	2.01 ± 1.50
Poultry with skin (4479)	2.09 ± 1.60
Other fatty cuts of meat (4476)	1.68 ± 1.63
Overall (4553)	1.85 ± 0.92

Tables 4 and 5 describe the regression results for associations between parental feeding styles and children’s dietary behaviour. After adjusting for parents' and children's demographic characteristics, ‘instrumental feeding’ was associated with lower likelihood of children consuming the recommended intake of vegetables (OR = 0.797; 95% C.I. = 0.662 to 0.961) and breakfast-eating frequency (OR = 0.767; 95% C.I. = 0.636 to 0.925), while children whose parents had higher scores in ‘emotional feeding’ were less likely to meet suggested intake of fruits (OR = 0.823; 95% C.I. = 0.712 to 0.952) and vegetables (OR = 0.815; 95% C.I. = 0.677 to 0.980). ‘Prompting and encouragement to eat’ was associated with higher chances of children meeting their suggested intake of fruits (OR = 1.357; 95% C.I. = 1.188 to 1.551), vegetables (OR = 1.335; 95% C.I. = 1.128 to 1.579) and dairy products (OR = 1.392; 95% C.I. = 1.210 to 1.602). Parents with higher scores in ‘Control over eating’ were more likely to have children who ate their recommended intake of fruits (OR = 1.533; 95% C.I. = 1.285 to 1.828) and vegetables (OR = 2.146; 95% C.I. = 1.709 to 2.695) and who ate breakfast every day (OR = 2.125; 95% C.I. = 1.709 to 2.643). However, their children had less chance of consuming their suggested intake of dairy products (OR = 0.736; 95% C.I. = 0.613 to 0.883). Additionally, ‘instrumental feeding’ (β = 0.216; 95% C.I. = 0.157 to 0.275) and ‘emotional feeding’ (β = 0.173; 95% C.I. = 0.116 to 0.230) were related to a higher consumption of high-ED foods, whereas ‘control over eating’ was associated with lower consumption in this category (β = -0.417; 95% C.I. = -0.485 to -0.349). The relationships remained significant for ‘instrumental feeding’ (β = 0.233; 95% C.I. = 0.175 to 0.291), ‘emotional feeding’ (β = 0.175; 95% C.I. = 0.119 to 0.231) and ‘control over eating’ (β = -0.390; 95% C.I. = -0.458 to -0.323) after adjusting for demographic characteristics.

**Table 4:** Logistic regression on associations between parental feeding styles and fulfilling recommended dietary intake or meal practice.

Parental feeding styles ^a^	Adjusted odds ratio ^b^ (95% confidence interval)			
	Fruits	Vegetables	Dairy products	Breakfast
Instrumental feeding	0.883	0.797	0.976	0.767
	(0.759,1.026)	(0.662,0.961)*	(0.834,1.141)	(0.636,0.925)*
Emotional feeding	0.823	0.815	1.080	1.149
	(0.712,0.952)*	(0.677,0.980)*	(0.929,1.256)	(0.961,1.373)
Prompting and Encouragement to Eat	1.357	1.335	1.392	1.065
	(1.188,1.551)*	(1.128,1.579)*	(1.210,1.602)*	(0.904,1.255)
Control over eating	1.533	2.146*	0.736	2.125
	(1.285,1.828)*	(1.709,2.695)	(0.613,0.883)*	(1.709,2.643)*

^a ^*:p-value <0.05

^b^ Odds ratios are adjusted for children’s gender, school grade, school status, place of birth, number of siblings; job status and education level of parents, household income per month, and relationship of respondent to the child.

**Table 5:** Linear regression on associations between parental feeding practice and the average consumption of high-ED food.

Parental feeding styles ^a^	Regression coefficients (95% confidence interval)	
	Average consumption of high-ED food	
	Crude	Adjusted ^b^
Instrumental feeding	0.216 (0.157, 0.275)*	0.233 (0.175, 0.291)*
Emotional feeding	0.173 (0.116, 0.230)*	0.175 (0.119, 0.231)*
Prompting and Encouragement to Eat	-0.012 (-0.064, 0.040)	-0.005 (-0.056, 0.046)
Control over eating	-0.417 (-0.485, -0.349)*	-0.390 (-0.458, -0.323)*

^a ^*:p-value <0.05

^b^ Regression coefficients are adjusted for children’s gender, school grade, school status, place of birth, number of siblings; job status and education level of parents, household income per month, and relationship of respondent to the child.

## Discussion

This has been the first large-scale cross-sectional study to investigate the association between parental feeding styles and dietary intake among pre-school students in Hong Kong. Parental feeding styles were assessed using a validated instrument (PFSQ), translated into Chinese. Information on pre-school children's consumption of fruit, vegetables, dairy products and high-ED foods, as well as their breakfast-eating patterns, was obtained from persons who were familiar with the children’s diets. Instrumental and emotional feeding was associated with unhealthy dietary pattern, while encouragement to eat healthily and control over food intake might relate to healthier food choices of children.

In the present study, although the majority of children ate breakfast every day, less than half of them consumed the recommended amounts of fruit, vegetables and dairy products. The problem is even more severe in the case of vegetables, as less than 20% of children consumed enough of them. Intervention strategies should be developed to change children’s unhealthy dietary patterns. As for high-ED foods, most children do not consume them in daily basis. However, there is still plenty of room to cut down the consumption.

Our study found that each parental feeding style made a different impact on school children's eating habit from early childhood. ‘Control over eating’ was associated with higher intake of fruits and vegetables, which correlated with lower consumption of high-ED foods and reduced chance of skipping breakfast. This phenomenon may be explained by the overt control assessed in the PFSQ. Overt control is defined as parental control that can be perceived by children,[28] such as telling children the types and quantities of food to eat. When compared with other feedings styles, children are more likely to meet the recommendations of the Department of Health if their parents exercise control over what they eat more frequently. In Hong Kong, both children and parents are well-educated by the Department of Health in the recommended intakes of fruits and vegetables, and the benefits of having breakfast every day, which enables parents to guide their children. Similarly, parents have learnt about the associations between high-ED foods and obesity, so they may limit their children's consumption in this area. However, children are less likely to consume dairy products in the recommended amounts if parents use ‘control over eating’, which is the only exception. One feeding style may not be enough to impart healthy eating behaviour in children. Also, children may have less ability to regulate their own diets if parents impose too much control over their food choices.[29] Nutritional education on the health effects of dairy products, such as promoting growth and development, should be emphasized, so that children may be less reluctant to adapt.

Similarly, children may have healthier dietary patterns when their parents use ‘prompting and encouragement to eat’ to influence their habits. The effectiveness of this type of influence in promoting healthy eating habit is similar to ‘control over eating’. In this survey, encouragement on eating was associated with the consumption of fruits, vegetables and dairy products, as well as breakfast-eating pattern. Parental encouragement was found to be associated with more frequent consumption of fruits and vegetables in children [9] and pre-school children,[10] but its effect on dairy food consumption and breakfast-eating frequency was firstly identified in our present research. Moreover, encouragement on eating contributed to greater consumption of dairy products, while control over eating did not. However, ‘control’ could reduce children’s consumption of high-ED food, unlike ‘encouragement’. There is a need to combine two parental feeding styles in modifying children’s dietary behaviour: ‘encouragement’ refers to parents offering more types of food but children can still make the decisions; while ‘control’ implies clearer instructions to children on their dining practices. In other words, the combination of two practices may resemble authoritative feeding style, because both parents and children have made contributions in determining food choices. [14] Authoritative feeding style has shown associations with more frequent consumption by children of fruits, vegetables and dairy products, [10,14] which should be an appropriate practice for parents. However, the present study has not examined how ‘encouragement’ and ‘control’ on eating might relate to authoritative feeding style. Interplay between various constructs of feeding styles should be investigated in future studies.

In contrast to the two previous types of feeding styles, instrumental feeding and emotional feeding were linked to less-favourable eating habits in children. Either one or both of them was related to inadequate consumption of fruit, vegetables and breakfast. In addition, both feeding styles showed a positive correlation with intake of high-ED foods. The detrimental effects of both feedings styles are consistent with similar research conducted before. [22] Parents may use high-ED food, which usually has a more appealing smell and taste than fruits and vegetables, as rewards for children’s behaviour or to relieve their emotion. Apart from reducing consumption of other, healthier food items, using unhealthy foods as rewards may also promote children’s preference for ‘rewarding’ food and hence a higher risk of overeating. [30] It is preferable to encouraging children to consume a wider variety of foods, as well as directing them towards the recommended dietary habits. Further studies are needed, in order to investigate whether instrumental and emotional feeding belongs to a distinct dimension of feeding style.

Our research has provided cues to investigate the correlations between children's eating habit and various constructs of feeding styles. Based on our findings, in order to promote healthy eating, parents should monitor the types of food provided to their children, as well as acting as role models. Also, parents need to encourage children to eat a variety of foods. In accordance with social learning theory, children are more likely to acquire healthy eating habits by imitating parental behaviour. However, parents should avoid feeding children according to their emotional needs or behaviour. Promoting children's health is the foremost consideration when selecting foods. By exploring more similarities among feeding styles in different dimensions, researchers may construct a more unified theoretical framework on parental influence over children’s dietary patterns.

The strength of the present project is its large sample size, when compared with previous research, which makes the findings more widely applicable. Although different impacts of feeding styles on dietary habit were observed, several limitations should be addressed in making a cautious interpretation. First of all, the dietary questionnaire only enquired about food consumption 7 days prior to the survey, which might not reflect a person's general dietary pattern. Also, the single-item question on the consumption of a specific food group might lead to underestimation of intake. Although photographs of food, as used in this survey, can improve the accuracy of dietary assessment, [31] the results should be confirmed by more comprehensive assessment method such as a food frequency questionnaire. Also, parents might not know the nature or amount of additional food provided to children when they were not under parental supervision, e.g. during tea-time in kindergarten; children's food consumption might be underestimated. In future studies, information supplied by other persons who look after the children, such as teachers, should be included when assessing children’s dietary pattern. In addition, the cross-sectional nature of the present project limited its conclusiveness on causality. A longitudinal study should be performed on the associations between parental feeding styles and children's dietary intake, as well as their weight status.

## Conclusion

The current study has observed the association between parental feeding styles and the dietary patterns of pre-school children from a reasonably large population. Instrumental and emotional feeding reduces the consumption of fruits, vegetables and breakfast, and promotes consumption of high-ED foods. Control and encouragement on food intake have the opposite impact. It is notable that no feeding style is adequate to promote healthy eating habits in children. However, the conclusiveness of our research is limited by the comprehensiveness of the dietary assessment method and cross-sectional design. Longitudinal research and behavioural interventions targeting parental eating habits are required to confirm the relationship.
